# Federated radiomics analysis of preoperative MRI across institutions: toward integrated glioma segmentation and molecular subtyping

**DOI:** 10.3389/fradi.2025.1648145

**Published:** 2025-11-10

**Authors:** Ran Ren, Anjun Zhu, Yaxi Li, Huli Liu, Guo Huang, Jing Gu, Jianming Ni, Zengli Miao

**Affiliations:** 1 Department of Radiology, Wuxi Ninth People's Hospital Affiliated to Soochow University, Wuxi, China; 2 Wuxi School of Medicine, Jiangnan University, Wuxi, China; 3 Department of Neurosurgery, Lianshui County People’s Hospital, Lianshui, China; 4 Clinical Internal Medicine Department, Shanghai Health and Medical Center, Wuxi, China; 5 Department of Neurosurgery, Jiangnan University Medical Center (JUMC), Wuxi, China

**Keywords:** federated learning, multi-institutional, multi-task deep learning model, molecular subtyping, image segmentation

## Abstract

**Background:**

Non-invasive and comprehensive molecular characterization of glioma is crucial for personalized treatment but remains limited by invasive biopsy procedures and stringent privacy restrictions on clinical data sharing. Federated learning (FL) provides a promising solution by enabling multi-institutional collaboration without compromising patient confidentiality.

**Methods:**

We propose a multi-task 3D deep neural network framework based on federated learning. Using multi-modal MRI images, without sharing the original data, the automatic segmentation of T2w high signal region and the prediction of four molecular markers (IDH mutation, 1p/19q co-deletion, MGMT promoter methylation, WHO grade) were completed in collaboration with multiple medical institutions. We trained the model on local patient data at independent clients and aggregated the model parameters on a central server to achieve distributed collaborative learning. The model was trained on five public datasets (*n* = 1,552) and evaluated on an external validation dataset (*n* = 466).

**Results:**

The model showed good performance in the external test set (IDH AUC = 0.88, 1p/19q AUC = 0.84, MGMT AUC = 0.85, grading AUC = 0.94), and the median Dice of the segmentation task was 0.85.

**Conclusions:**

Our federated multi-task deep learning model demonstrates the feasibility and effectiveness of predicting glioma molecular characteristics and grade from multi-parametric MRI, without compromising patient privacy. These findings suggest significant potential for clinical deployment, especially in scenarios where invasive tissue sampling is impractical or risky.

## 1 Introduction

Gliomas are the most frequent primary brain tumors, exhibiting substantial prognostic and therapeutic variability due to molecular heterogeneity (1). Key molecular biomarkers including isocitrate dehydrogenase (IDH) mutation, 1p/19q chromosomal co-deletion, O6-methylguanine-DNA methyltransferase (MGMT) promoter methylation, and WHO tumor grade significantly influence clinical outcomes and treatment response. Accurate, non-invasive characterization of these biomarkers is increasingly essential for personalized therapeutic decision-making, prognostic assessments, and treatment monitoring. Traditionally, molecular profiling has relied on invasive surgical resections or biopsies, procedures that carry inherent risks such as hemorrhage, infection, and neurological damage, and can be impractical due to tumor location or patient frailty. Additionally, biopsy-derived molecular analyses are susceptible to sampling bias, potentially failing to fully capture the heterogeneous nature of gliomas and limiting the accuracy and representativeness of molecular characterization (2–4).

Recent advancements in radiomics and deep learning have facilitated the development of non-invasive molecular subtyping methods through MRI-based computational analyses, significantly enhancing the clinical utility of imaging data (5). MRI offers comprehensive anatomical and functional information through a variety of sequences, including pre- and post-contrast T1-weighted, T2-weighted, and fluid-attenuated inversion recovery (FLAIR), enabling detailed insights into tumor morphology and physiology. Deep convolutional neural networks (CNNs), known for their powerful capacity to extract and interpret high-dimensional hierarchical imaging features, have demonstrated remarkable efficacy in accurately predicting molecular biomarkers and delineating tumor boundaries (6). However, conventional methods predominantly utilize single-task models that focus either on predicting isolated molecular markers or exclusively performing tumor segmentation, without exploiting the potential synergies and shared biological contexts among these tasks. This approach not only limits the depth of clinical insights achievable from imaging data but also reduces the interpretability and integrative clinical applicability of predictive outcomes.

To overcome the limitations of single-task models, an important research trend is the development of unified frameworks capable of performing both classification and segmentation simultaneously. Such multi-task learning (MTL) approaches can leverage shared feature representations, allowing the spatial localization information provided by the segmentation task to support the classification task, thereby enhancing the overall performance and interpretability of the model. For example, previous studies (7, 8), which are highly relevant to this work, have successfully designed advanced single models that can simultaneously predict diagnostic labels and precisely delineate tumor boundaries, often employing methods such as Grad-CAM to visualize the association between the model’s classification decisions and specific tumor subregions.Although these powerful multi-task models have set new benchmarks for comprehensive glioma analysis, they generally rely on large-scale, high-quality centralized datasets.In practical applications, centralized training models face substantial challenges such as stringent data-sharing restrictions due to regulatory frameworks, diverse imaging protocols, and site-specific variability in patient populations (9). Such factors result in restricted model generalizability, hindering broader clinical adoption and scalability.

FL has recently emerged as an innovative methodology to tackle these critical challenges, allowing distributed model training across multiple institutions without sharing patient-level data. FL aggregates model parameters rather than raw data, ensuring compliance with privacy standards and ethical guidelines, while improving the diversity and generalization capability of trained models across heterogeneous populations (10).In the context of federated learning, existing studies have primarily focused on three types of single tasks: image segmentation, classification, and image-to-image translation. In segmentation, efforts aim to enhance generalization and structural modeling. Alphonse et al. proposed an attention-based multiscale U-Net under a federated framework for high-precision tumor segmentation on the BraTS dataset (11). Zhou et al. introduced Fed-MUnet to improve cross-modal consistency in multi-modal MRI (12), while Bercea et al. developed FedDis, sharing only shape encoder parameters to support weakly supervised lesion segmentation (13). Manthe et al. established a BraTS federated benchmark to evaluate aggregation strategies systematically (14).For tumor classification, FL enables robust subtype prediction across centers. Ali et al. combined 3D CNN and focal loss in the EtFedDyn framework to jointly predict IDH mutation and WHO grading, achieving centralized-level performance (15). Mastoi et al. enhanced model interpretability via gradient-weighted class activation mapping (Grad-CAM) (16), and Gong et al. tackled non-IID challenges through perturbation-based aggregation.In image-to-image translation (17), Wang et al. introduced FedMed-GAN, integrating GANs into FL for unsupervised MRI modality synthesis (18). Fiszer et al. benchmarked ten FL strategies for multi-contrast MRI translation (19), and Al-Saleh et al. developed a federated GAN model combining synthesis and segmentation with privacy protection (20). Notably, a recent study by Raggio et al. represents a significant breakthrough by being the first to successfully generate synthetic CT from MRI within a global, multi-center, distributed training framework (21). Their model demonstrated robust generalization on an external validation cohort from a center not included in the original federation. This work provides strong evidence for the advantages of federated learning in enabling privacy-preserving, cross-institutional image synthesis with excellent generalizability. The fact that their study utilized several variants of the U-Net architecture aligns closely with our own choice of a U-Net-based structure for our model. Overall, FL has demonstrated strong capabilities in segmentation, classification, and translation, advancing collaborative modeling. However, as mentioned above, although centralized multi-task learning has made progress, most current federated learning studies still focus on single-task modeling, such as performing only segmentation or classification, and usually rely on similar modalities and label structures. In real-world applications—such as glioma analysis—tasks like segmentation, grading, and molecular subtyping often co-exist, with heterogeneous or incomplete annotations across institutions. Therefore, a key challenge that remains to be addressed is how to combine the advantages of multi-task learning in terms of performance and interpretability with the strengths of federated learning in privacy preservation and generalization, while adapting to real clinical demands such as label heterogeneity and modality robustness.

To align with this research direction and address the above challenges, this study proposes a federated multi-task learning framework for glioma analysis. It enables collaborative training of models across multiple medical centers using preoperative MRI data without sharing raw data, jointly predicting the IDH mutation status, 1p/19q co-deletion status, MGMT methylation status, tumor grading, and automatically segmenting the T2-weighted hyperintense tumor region. Our framework uniquely combines federated learning—a decentralized, privacy-preserving training approach—with an advanced 3D convolutional neural network architecture optimized for comprehensive analysis of multi-parametric MRI data. Specifically, our model simultaneously predicts critical glioma biomarkers including IDH mutation, 1p/19q co-deletion, MGMT promoter methylation, and WHO tumor grade (II, III, IV), while precisely delineating the tumor's T2-weighted hyperintense region. Importantly, our federated strategy facilitates collaboration across multiple institutions without sharing patient-level data, significantly enhancing data diversity and model generalization. Additionally, we incorporated Grad-CAM interpretability into our architecture, enabling visualization of spatial attention patterns and providing clinical experts with transparent insights into the model's decision-making processes. To our knowledge, this represents the first implementation integrating federated learning with multi-task CNNs and interpretability tools in glioma imaging, offering both robust clinical predictions and enhanced transparency for clinical adoption. Leveraging the computational capabilities of state-of-the-art GPUs, optimizing memory consumption, and employing distributed federated multi-institutional training, our model efficiently processes entire 3D MRI volumes. Training was performed on a diverse patient cohort comprising 1552 patients across five publicly available datasets (BraTS2021, UPENN-GBM, REM- BRANDT, TCGA-GBM, and TCGA-LGG) from multiple institutions. To maximize clinical relevance and applicability, minimal inclusion criteria were applied—requiring only the four standard MRI sequences: pre- and post- contrast T1-weighted, T2-weighted, and T2-FLAIR (22). No exclusion criteria based on clinical characteristics (e.g., tumor grade) or radiological quality (e.g., scan artifacts) were imposed, thereby capturing the intrinsic heterogeneity representative of routine clinical practice. The generalizability of our method was rigorously evaluated on an independent dataset comprising 466 patients from external multi-institutional cohorts, confirming robust performance and broad applicability across diverse clinical settings.

## 2 Materials and methods

### 2.1 Patient population

Our study is based on retrospectively collected data from five publicly available datasets: BraTS2021 (23), UPENN-GBM (24), TCGA-GBM (25), TCGA-LGG (25), and REMBRANDT. The first dataset, UPENN-GBM, was sourced from the University of Pennsylvania Health System and includes multi-parametric magnetic resonance imaging (mpMRI) scans of 671 newly diagnosed glioblastoma (GBM) patients. These images underwent standardized preprocessing procedures, including skull-stripping and co-registration. The second dataset, BraTS2021, was used after excluding any overlapping patients from the TCGA-LGG, TCGA-GBM, and UPENN-GBM subsets to avoid data leakage.

Manual segmentation annotations were available in the BraTS and UPENN-GBM datasets. The BraTS dataset employed the training and validation cohorts from the 2021 BraTS challenge, which included manually segmented tumor regions. In the UPENN-GBM dataset, tumor subregion labels were generated via computer-assisted annotation followed by manual corrections. These segmentations were created by a diverse set of qualified raters, introducing heterogeneity in annotation styles.

Patients were included if they were newly diagnosed with glioma and had available pre- and post-contrast T1- weighted (T1w), T2-weighted (T2w), and T2w-FLAIR scans. No additional exclusion criteria were applied based on radiologic quality (e.g., low image resolution or artifacts) or clinical features (e.g., tumor grade). In cases where multiple scans of the same modality were available for a patient (e.g., multiple T2w scans), the scan used for segmentation was selected. If no segmentations were available or if segmentations were not derived from the specified modality, the scan with the highest axial resolution was chosen, with preference given to 3D acquisitions over 2D.

Meanwhile, we utilized the TCGA-LGG and TCGA-GBM collections from TCIA, as well as the REMBRANDT collection from the Molecular Brain Tumor Data Repository. Both the TCGA-LGG and TCGA-GBM datasets include multi-modal MRI scans, comprising T1, T1CE, T2, and FLAIR sequences.The original images are mainly three-dimensional (3D) MRI, but due to multi-center acquisition, the images have some differences in spatial resolution, slice thickness and contrast consistency. In addition, some images have slight motion artifacts or low signal-to-noise ratio problems.Genetic and histological annotations were collected from both TCIA clinical records and the dataset published by Ceccarelli et al. Segmentation masks for these cohorts followed the BraTS 2021 Challenge guidelines, with tumors manually delineated by one to four annotators and reviewed by board-certified neuroradiologists. The inclusion criteria for these patients mirrored those of our training cohort, requiring pre- and post-contrast T1w, T2w, and T2w-FLAIR scans.Genomic and histopathological metadata were available for public datasets. For REMBRANDT, clinical and molecular data were obtained from TCIA; for UPENN-GBM, these were collected from the clinical archives of the University of Pennsylvania Health System. The BraTS2021 dataset was sourced directly from the 2021 BraTS Challenge.

There are some differences in imaging protocols for different datasets. For example, the BraTS and UPENN-GBM datasets are both high-quality three-dimensional acquisition (3D MRI), whereas the REMBRANDT data is 3D but has wide variation in slice thickness and image quality. In terms of scanning sequence, each dataset contained T1, T1CE, T2 and FLAIR modes, but there were inconsistencies in spatial resolution, slice spacing, and whether it was 2D or 3D acquisition. In addition, the images in UPENN and REMBRANDT come from different hospitals or scanners, and there may be variation in magnetic field strength and noise level. At the annotation level, BraTS and UPENN provide accurate manual segmentation, while the REMBRANDT dataset is mainly used for classification analysis due to the lack of pixel-level segmentation information.It should be noted that although most TCGA cases had complete four-modality MRI and high-quality segmentation signatures, some cases were not included in the analysis due to missing or image quality problems on specific sequences such as T1 or FLAIR.

Three patients were excluded from the final evaluation cohort due to not meeting these imaging requirements despite the availability of manual annotations: TCGA-08-0509 and TCGA-08-0510 from TCGA-GBM lacked precontrast T1w scans, and TCGA-FG-7634 from TCGA- LGG lacked post-contrast T1w imaging.

### 2.2 Patient characteristics

A total of 1,552 patients were included in this study, with 1,086 (approximately 70%) assigned to the training set and 466 (approximately 30%) to the testing set. Patient characteristics of the training set and the test set are shown in Table 1, including gender, age, IDH status, 1p/19q co-deletion status, MGMT methylation status, and tumor grade. In the training set, the proportion of males and females was 55.52% and 38.12%, respectively. The age was mainly concentrated in people aged 40–60 years old and over 60 years old (about 76%). The distribution of the test set was consistent with the training set. IDH mutation rate was low (about 3–4%) and 1p/19q co-deletion rate was about 50% in both sets. MGMT methylation status showed similar trends in different sets. About 31% of the patients had grade IV gliomas, and the remaining patients were mainly distributed in grade II and III, but some cases lacked complete grading information.

**Table 1:** Characteristics of the patients in the training and test sets.

Characteristic	Train Set		Test Set	
	*N*	%	*N*	%
Total	1,086	100	466	100
Sex				
Female	414	38.12s%	192	41.20%
Male	603	55.52%	257	55.15%
Unknown	69	6.35%	35	7.51%
Age(years)				
<40	100	9.21%	41	8.80%
40–60	358	32.97%	154	33.05%
>60	469	43.19%	202	43.35%
Unknown	159	14.64%	69	14.81%
IDH status				
Mutated	41	3.78%	16	3.43%
Wildtype	716	65.93%	308	66.09%
Unknown	329	30.29%	142	30.47%
1p/19q co-deletion status				
Co-deleted	184	16.94%	79	16.95%
Intact	545	50.18%	233	50.00%
Unknown	357	32.87%	154	33.05%
MGMT status				
Unmethylated	210	19.34%	89	19.10%
Methylated	263	24.22%	113	24.25%
Unknown	613	56.45%	264	56.65%
Grade				
II	61	5.62%	27	5.79%
III	60	5.52%	26	5.58%
IV	338	31.12%	146	31.33%
Unknown	627	57.73%	267	57.30%

To quantify dataset heterogeneity, we compared key characteristics across the five public datasets used in this study (BraTS2021, UPENN-GBM, TCGA-GBM, TCGA-LGG, and REMBRANDT), as shown in Table 2. A total of 1,552 cases were included, with notable differences in WHO grade distribution: lower-grade tumors (Grade II/III) were more prevalent in TCGA-LGG and REMBRANDT, whereas BraTS2021, UPENN-GBM, and TCGA-GBM were predominantly high-grade (Grade IV) tumors.

**Table 2:** Heterogeneity across the datasets.

Dataset	Cases (*n*)	WHO Grade II	WHO Grade III	WHO Grade IV	IDH mutation (*n*, %)	1p/19q codeletion (*n*, %)	MGMT promoter methylation (*n*, %)
BraTS2021	384	0	4	329	11 (2.86%)	218 (56.77%)	255 (66.41%)
UPENN-GBM	671	0	0	132	19 (2.83%)	19 (2.83%)	121 (18.03%)
TCGA-GBM	262	0	0	0	5 (1.91%)	0	0
TCGA-LGG	108	47	59	0	22 (20.37%)	26 (24.07%)	0
REMBRANDT	127	41	25	23	0	0	0
**Total**	1,552	88	88	484	57 (3.67%)	263 (16.95%)	376 (24.23%)

Percentages indicate the proportion of cases with each molecular marker or complete modality relative to the total number of cases in the dataset.

The distribution of molecular markers also varied substantially. IDH mutation was relatively common in BraTS2021 (2.86%) and TCGA-LGG (20.37%), but rare in UPENN-GBM (2.83%) and TCGA-GBM (1.91%). A similar pattern was observed for 1p/19q codeletion, which was enriched in TCGA-LGG (24.07%) and BraTS2021 (56.77%), but nearly absent in high-grade GBM datasets. MGMT promoter methylation also showed marked differences across datasets, with the highest proportion in BraTS2021 (66.41%) and the lowest in UPENN-GBM (18.03%).

These results demonstrate substantial variability in tumor grade and molecular characteristics across datasets, highlighting the necessity of using federated learning to aggregate multi-center data without sharing raw images. They also underscore the applicability of the multi-task model for handling diverse patient populations and molecular subtypes.

### 2.3 Federated learning framework

To achieve collaborative modeling across health care organizations and avoid patient data leakage, we adopted a FL strategy. Unlike the classical FedAvg framework, which aggregates models by exchanging parameters, we propose an innovative strategy based on knowledge distillation and compressed data transmission, aiming to reduce communication overhead and enhance model fusion. This strategy enables multiple hospitals (clients) to collaboratively train a global model without sharing raw images or model parameters.

In this study, an innovative federated learning framework is proposed to efficiently complete the tasks of medical image analysis and classification under the premise of ensuring the data privacy of medical institutions. The framework gradually optimizes the shared global model through the cooperation of the client and the server. The client-side procedure ensures that raw patient data is neither directly used for model training nor transmitted. A process, termed knowledge refining, is first employed to transform the local data into a condensed and abstract representation. This type of compressed data is a compact and information-dense representation that is able to preserve key medical features in the original data as much as possible without compromising privacy. The compressed data generation process is based on a Compression loss: the loss function is optimized so that the output of the model when processing the compressed data is as close as possible to its performance when processing the original data, such as the extracted features or the predicted classification probability. Specifically, the client compares the prediction of the model on the compressed data with its performance on the local real data to guide the synthesis of the compressed data. This mechanism ensures that the compressed data effectively represent the original information in a medical sense. The client only uploads the generated compressed data, and there is no need to share the original image or patient information, which protects the privacy and greatly reduces the communication overhead.

On the server side, the process of aggregation and training is particularly critical. The server integrates the compressed data from each client and uses the two objective functions to jointly optimize the global model parameter *θ*. Firstly, the Cross-entropy loss is used for basic supervised learning to ensure that the model can accurately classify the compressed data. Second, Distillation matching loss is introduced to further mine and integrate the deep knowledge from different clients. This loss function works by comparing two types of Soft labels: the first component is the prediction of the current global model on the compressed data, which reflects the model's interpretation of that data; the other category is soft labels calculated and uploaded by the client on local real data, representing the knowledge refined by the client. Different from the traditional hard labels (single category label), soft labels express the confidence level of the model for each category in the form of probability distribution and contain more fine-grained information. By minimizing the difference between the server-side prediction soft label and the client-side soft label, the distillation match loss realizes the migration from the client's real data knowledge to the global model, effectively alleviates the problem of information loss that may be caused by compressed data, and improves the stability and generalization ability of the model.Within this federated framework, we introduce a multi-task deep learning model based on the U-Net architecture, designed to simultaneously perform tumor region segmentation as well as genetic and histopathological feature prediction. This model serves as the global model to be optimized on the server side and as the foundational architecture for local models on the client side, which are used to generate compressed data and soft labels.

Finally, the server combines the cross-entropy loss with the distillation matching loss to obtain a global model with more generalization ability and robustness. The model was then distributed to the client, leading to a new round of more accurate data compression and knowledge extraction. Through continuous iteration, the whole federated learning process is continuously optimized, and finally converges to a medical image analysis model with stable performance and perfect privacy protection.

### 2.4 Classification model

**Figure 1:**
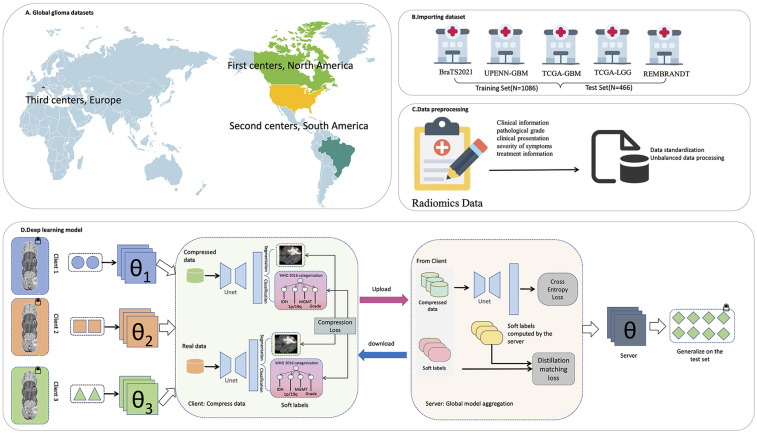
The overall framework of the research. **(A)** Global glioma dataset distribution. **(B)** Import the dataset. **(C)** Data preprocessing. **(D)** Deep learning model.

To realize the proposed privacy-preserving federated knowledge distillation framework, a powerful and efficient deep learning model forms the core. This model not only serves as the final global model to be optimized on the server side but also functions as the tool for each client to generate compressed data and soft labels locally. Within this federated framework, we introduce a multi-task model based on the U-Net architecture, designed to simultaneously perform tumor region segmentation and predict genetic and histopathological features (IDH, MGMT, 1p/19q, and Grade). The architecture is optimized for memory efficiency, enabling the use of entire 3D MRI scans as input to enhance spatial contextual modeling. The overall structure of the model is illustrated in Figure 1.

The encoder part consists of three downsampling stages, each consisting of two layers of 3D convolutions (convolution, batch normalization, and ReLU activation). The downsampling is implemented by 3D maximum pooling of size (1, 2, 2) to reduce planar resolution while preserving slice dimension. The decoder adopts a symmetric structure, which is gradually upsampled by transposed convolution and combined with corresponding encoder features to enhance spatial detail recovery. After the bottleneck layer, the network was divided into two output branches. The segmentation branch generated voxellevel segmentation prediction by 1 × 1 × 1 convolution and output four types of tumor regions. The classification branch extracted the global information from the bottleneck features, and then input the multi-layer fully connected network after global average pooling to predict four clinical indicators. Each task corresponding to an independent classification head (IDH mutation, 1p/19q co-deletion, MGMT methylation as a binary classification, and tumor grade as a triple classification).

In order to reduce the memory consumption and maintain the input consistency, all MRI images were registered to the standard atlas, skull stripping and intensity normalization in the preprocessing stage, and uniformly resampled and trimmed to a 128 × 128 planar resolution. All slices of the case were stacked in order to form a 3D volume, and the depth was determined according to the actual number of slices of the patient. During training, the full 3D volume was directly input to the model instead of local patches to fully retain the global spatial context information. The baseline convolutional channel number of U-Net is set to 16 (traditional 32), and it is multiplied sequentially in each down-sampling stage (16→32→64→128) to reduce the video memory footprint while maintaining the modeling ability. The input data contained four MRI modalities (T1, T1ce, T2, and FLAIR).

During model training, 20% of the training cohort was held out as a validation set for model selection. The final model was retrained on the entire training set using the selected hyperparameters and subsequently evaluated only once on an independent test set to ensure unbiased generalization and realistic performance estimation.

The model was trained using stochastic gradient descent (SGD) with an initial learning rate of 0.001 for a total of 60 epochs. The cross-entropy loss function was employed to quantify the discrepancy between the predicted outputs and the ground truth labels. All experiments were conducted on an NVIDIA RTX A6000 GPU, leveraging its high computational power to accelerate convergence. The implementation was developed using PyTorch 2.4.1, ensuring efficient training and scalability.

Model performance was quantitatively evaluated using the area under the receiver operating characteristic curve (AUC), accuracy, recall, F1 score, and specificity for molecular and histopathological predictions. To assess the model's segmentation capability, we further conducted visual analyses of attention maps derived from activation heatmaps, illustrating the regions most influential to the network's predictions.

### 2.5 Ethical considerations and data privacy

MRI data used in this study were obtained from publicly available datasets, including BraTS2021, UPENN-GBM, TCGA-GBM, TCGA-LGG, and REMBRANDT. These data sets were rigorously de-identified before publication and had received ethical approval from their organizing institutions, thus eliminating the need for additional informed patient consent.

In addition to this, the federated learning framework adopted in this study provides a higher level of privacy protection by design. In this simulated multicenter study, a core principle of federated learning was strictly observed: raw MRI data remained on local clients at all times and were never transmitted to a central server or any third party. Throughout the collaborative training process, participants shared only anonymized, aggregated model parameters (e.g., network weights) with a central server that did not contain any identifiable individual patient information. The reverse derivation of highly complex raw medical imaging data from these aggregated parameters is not feasible under the current technology conditions, which fundamentally ensures the confidentiality of patient data. The data use and methodological design of this study were designed to comply with the ethical guidelines of the Declaration of Helsinki and the spirit of data protection regulations such as the General Data Protection Regulation (GDPR) and the Health Insurance Portability and Accountability Act (HIPAA), providing a compliance basis for future deployment of this technology in real-world clinical Settings.

## 3 Results

### 3.1 Algorithm performance

**Figure 2:**
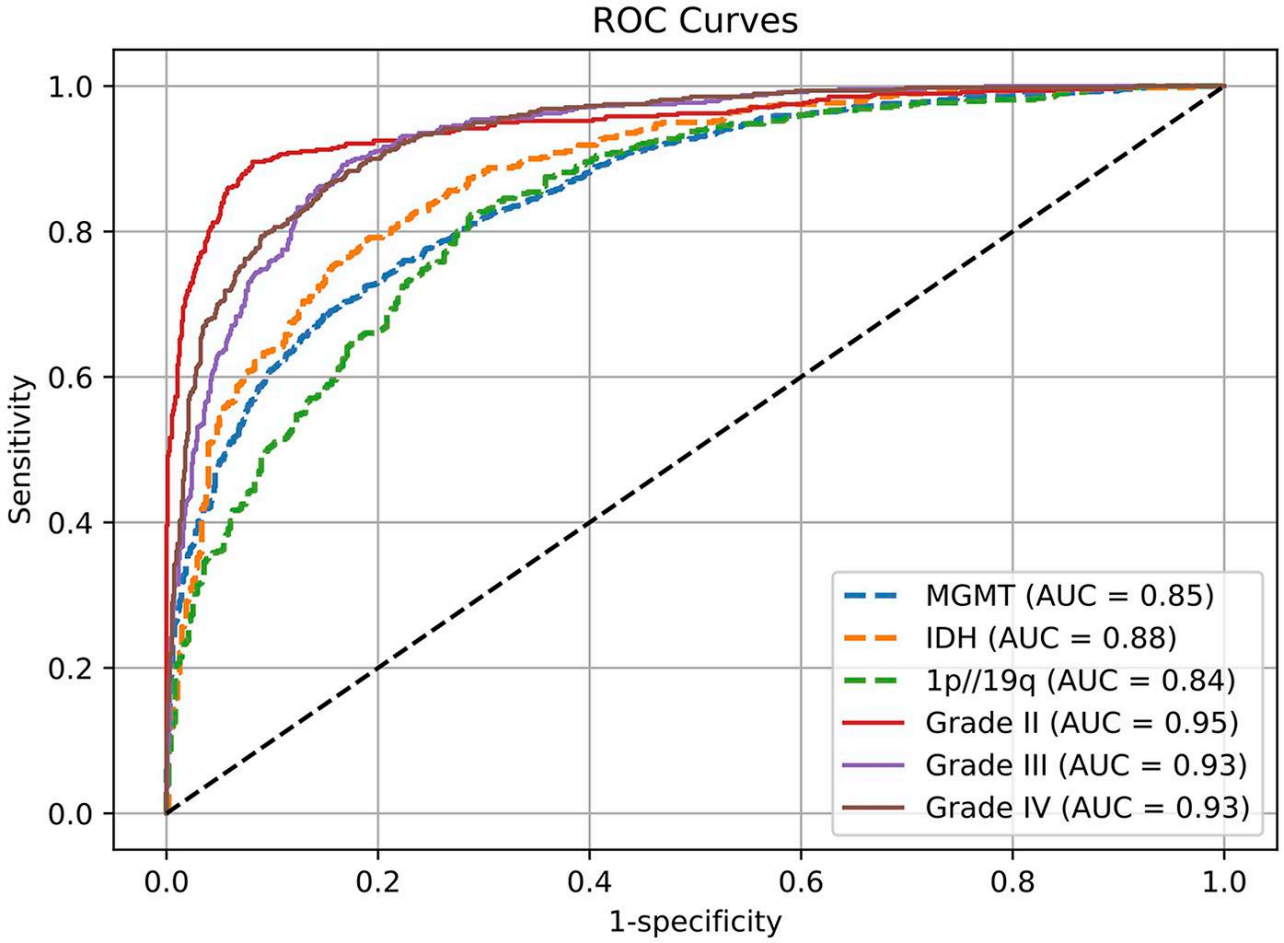
Receptor operating characteristic (ROC) curves for evaluating genetic and histological features on the test set.

We evaluated the model's performance on multiple glioma molecular subtype and tumor grading classificationtasks using the independent test set. The quantitative results are summarized in Table 3, and the corresponding receiver operating characteristic (ROC) curves are illustrated in Figure 2. Overall, the model demonstrated strong discriminative capability and robustness across all classification tasks, highlighting the effectiveness of multimodal information fusion for fine-grained glioma characterization (26).

**Table 3:** Evaluation results of the final model on the test set.

Task	AUC	ACC	Recall	SPEC	F1 Score
IDH	0.88 (95% CI: 0.810–0.860)	0.9	0.87	0.92	0.93
MGMT	0.85 (95% CI: 0.858–0.901)	0.86	0.86	0.87	0.86
1P/19Q	0.84 (95% CI: 0.838–0.868)	0.94	0.93	0.96	0.93
GRADE	0.94 (95% CI: 0.927–0.947)	0.91	0.94	0.93	0.9
GRADE II	0.95 (95% CI: 0.949–0.962)	0.96	0.98	0.97	0.95
GRADE III	0.94 (95% CI: 0.924–0.950)	0.93	0.95	0.96	0.93
GRADE IV	0.93 (95% CI: 0.929–0.944)	0.85	0.89	0.88	0.84

In the molecular subtype classification tasks, the model achieved an AUC of 0.88, accuracy of 0.90, and F1 score of 0.93 in predicting IDH mutation status, indicating high robustness in identifying this clinically critical biomarker. For 1p/19q codeletion status, although the AUC was relatively lower at 0.84, the model yielded excellent results in terms of accuracy (0.94) and recall (0.93), suggesting a low false-negative rate and strong sensitivity for detecting positive cases.

Prediction performance for MGMT promoter methylation was slightly lower compared to other molecular markers, with an AUC of 0.85, accuracy of 0.86, and F1 score of 0.86. This modest performance may be attributed to the limited number of MGMT-labeled samples in the training set or intrinsic modality-specific feature disparities. Nonetheless, the results remain clinically relevant and demonstrate practical utility.

For the tumor grading task, the model exhibited excellent overall performance, achieving an AUC of 0.94 and accuracy of 0.91 for predicting overall tumor grade, indicating its ability to effectively integrate multimodal information for accurate grading. Subtype-specific analysis further revealed outstanding performance in distinguishing Grade II and Grade III gliomas, with AUCs of 0.95 and 0.94, and F1 scores of 0.95 and 0.93, respectively. The performance on Grade IV tumors was comparatively lower (AUC = 0.93, F1 = 0.84), which may be due to greater intratumoral heterogeneity and complex imaging phenotypes associated with high-grade gliomas. Future work may consider incorporating attention mechanisms or hierarchical classification strategies to further enhance performance on this subgroup.

To validate the effectiveness of our approach, we conducted a comparative analysis using different combinations of input modalities, including: (1) T1 alone, (2) T1 combined with T2, (3) a three-modality combination of T1,T2, and T1ce, and (4) a fully integrated multimodal set- ting incorporating T1, T2, T1ce, and FLAIR. On a fixed test set, we evaluated the classification performance using area under the ROC curve (AUC), accuracy, recall, F1 score, and specificity for the prediction of molecular and histopathological features.

**Figure 3:**
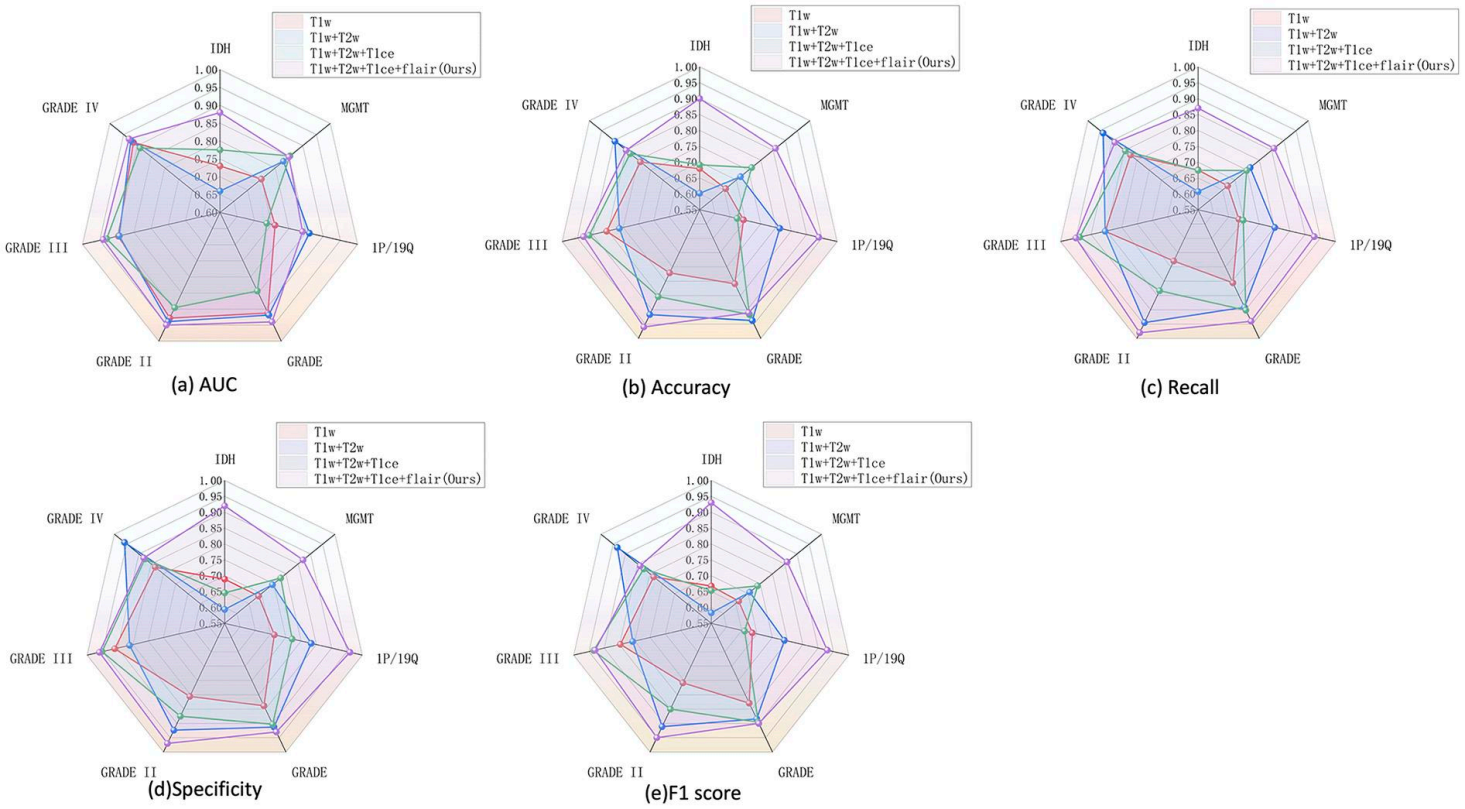
Performance of multimodal combination in molecular grading and classification of glioma. **(a)** AUC, **(b)** accuracy, **(c)** recall, **(d)** specificity, and **(e)** F1 score.

The results demonstrated that the multimodal configuration achieved the best performance across all evaluation metrics. Figure 3 presents a comprehensive comparison of five performance metrics (AUC, accuracy, recall, specificity, and F1 score) for four glioma-related classification tasks (IDH mutation, MGMT methylation, 1p/19q co-deletion, and tumor grade), systematically assessing the impact of different modality combinations. A consistent upward trend in classification performance was observed as more modalities were incorporated, particularly with the full four-modality combination (T1 + T2 + FLAIR + T1ce), which led to significant improvements in performance and model stability. These findings underscore the value of multimodal inputs in enhancing the model's generalization and representation capabilities.

Notably, the tumor grading task (GRADE) was the most sensitive to modality variation, with substantial gains observed when more modalities were fused. Clear improvements were also seen in IDH and 1p/19q predictions, while MGMT classification showed more modest gains, potentially due to the molecular feature's weaker radiographic representation or sample imbalance.When modality information is insufficient, the model struggles to learn robust features for minority classes, making its performance more vulnerable to the negative effects of class imbalance. In contrast, full-modality input provides richer feature dimensions, enabling the model to effectively overcome this issue and serving as our primary data-driven strategy to address the challenge. In terms of metric comparison, AUC and F1 score best capture the overall performance improvement brought by modality fusion, whereas specificity shows greater fluctuations in certain tasks (e.g., MGMT). This further confirms that the model exhibits instability in recognizing the negative (majority) class when dealing with imbalanced data.

Importantly, different modality combinations exhibited task-specific advantages. The full modality configuration (T1 + T2 + FLAIR + T1ce) achieved the highest AUC and F1 scores for IDH and GRADE classification tasks, demonstrating excellent discriminative capability and predictive robustness. In particular, for the multi-class tumor grading (GRADE II/III/IV), this configuration substantially improved recall and specificity, highlighting its effectiveness in modeling grade-associated anatomical characteristics. Meanwhile, the three-modality combination (T1 + T2 + FLAIR) performed competitively in the 1p/19q classification task, even outperforming T1ce-inclusive combinations in certain metrics, suggesting that this molecular feature may rely more heavily on macrostructural tumor morphology than on contrast-enhanced details. Overall, the T1 + T2 + FLAIR combination serves as a strong and robust baseline, while the inclusion of T1ce—when available—further boosts the model's multitask adaptability and classification accuracy, especially for tasks involving tumor boundary delineation and core structure characterization.In addition to using full modal data, algorithm-level strategies such as Focal Loss or weighted sampling can also be explored in the future to further optimize the performance of the model on imbalanced data.

**Figure 4:**
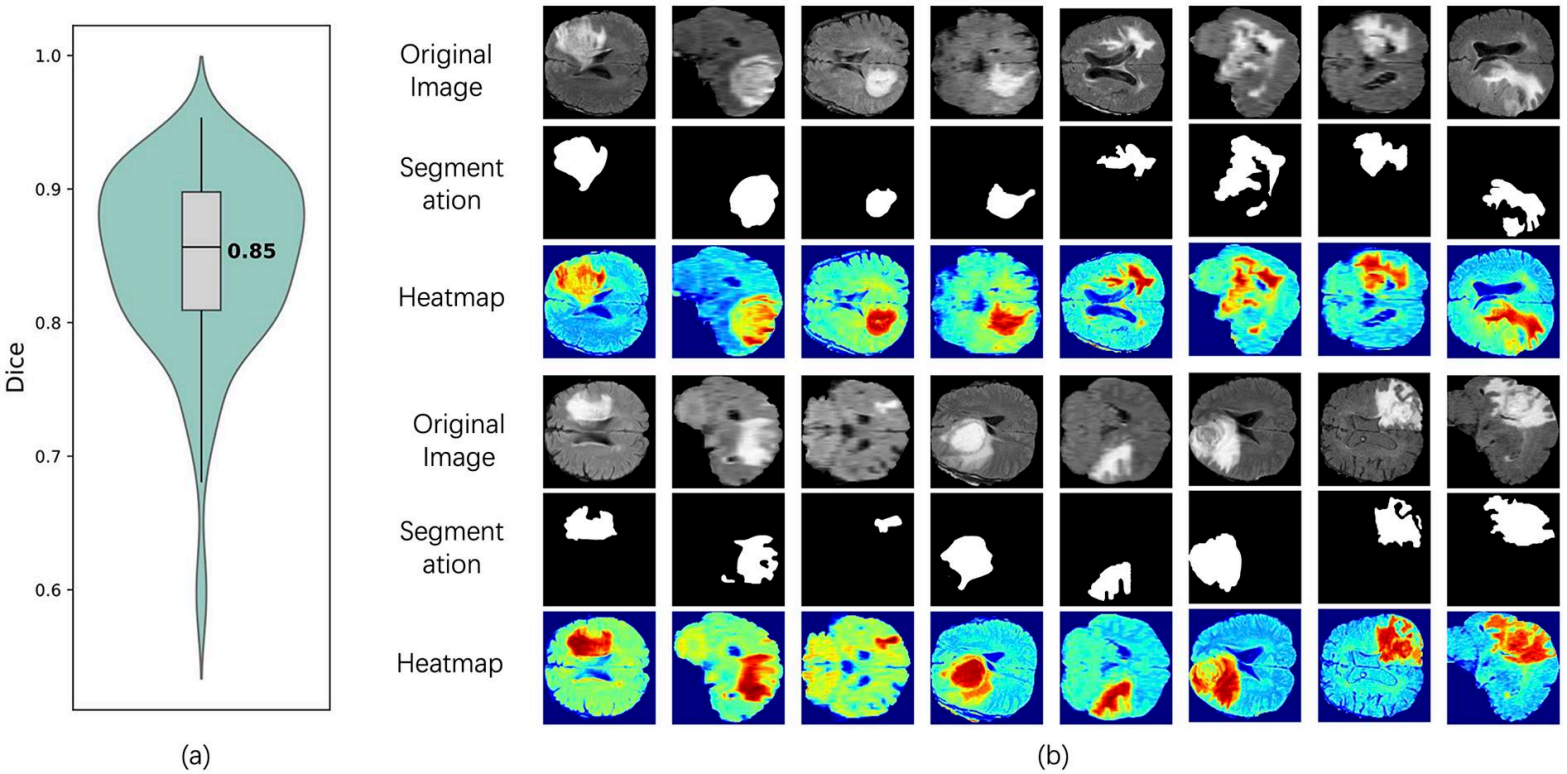
Segmentation performance and interpretability analysis of the model on the test set. **(a)** Dice coefficient violin plot of the segmentation results, showing the distribution of model performance on all tested samples, with a median of 0.85. **(b)** shows the original images of typical patients in the test set, the segmentation results of the model, and the interpretable heat map based on Grad-CAM. The red area represents the significant regions that the model focuses on when making segmentation judgments.

To comprehensively evaluate the model's segmentation performance in identifying T2w hyperintense regions, we combined both quantitative metrics and visual interpretability techniques. As shown in Figure 4a, the violin plot of Dice coefficients across all test samples demonstrates that the segmentation branch achieves stable performance with a median Dice score of 0.85, indicating accurate delineation of lesion boundaries from normal tissue. In addition, to further investigate the model's decision-making process and interpretability, we employed visual heatmap-based interpretability techniques (27), such as Grad-CAM (28). Figure 4b shows the visualization results of multiple representative patients in the test set, including the original image, the segmentation mask generated by the model and the corresponding Grad-CAM heat map. The segmentation results clearly marked the lesion area, and the spatial location was highly consistent with the high-activation area (red area) in the heat map, which further verified the accuracy of the model in extracting key features in complex brain anatomical structures. Compared with the traditional evaluation method that relies on pixel-level labels, the heatmap not only reveals the regions of concern for the segmentation decision of the model, but also provides an intuitive and interpretable reference for the performance of the model when there is a lack of accurate annotation, thereby improving the credibility of the model in practical clinical applications.

### 3.2 Model interpretability

To provide deeper insight into the model's behavior, heatmaps and selected encoder feature outputs were generated and visualized in Figure 4b.These visualizations highlight the regions within the scans that contributed most significantly to the model's predictions. The heatmaps revealed that the network predominantly focused on the hyperin- tense boundary regions in the T2w-FLAIR images and the strongly enhanced areas in post-contrast T1w scans during inference.

The heatmap in Figure 4b was generated using Grad-CAM to visualize the model's region-of-interest. its generation principle is explained as follows. Grad-CAM generates an interpretable localization map by analyzing the gradient information in the deep layers of the network. Specifically, the method begins by computing the gradients of the target category's score with respect to the feature maps of the final convolutional layer. These gradients reflect the importance of each feature channel for the ultimate decision. Subsequently, global average pooling is applied to these gradients to derive a set of weights, each representing the importance of a corresponding feature map. Finally, a weighted linear combination of the feature maps is calculated using these weights and then passed through a ReLU activation function to generate the final heatmap.In the resulting visualization, brighter regions indicate a greater contribution from the features in that area to the model's prediction. Thus, the heatmap in Figure 4b is not a simple image overlay but rather a visual representation of the model's internal decision-making process. It explicitly highlights which parts of the image were most influential in reaching the final prediction.

The visualization of encoder feature maps and segmentation results further showed that the model could effectively focus on the lesion area and accurately segment the structure consistent with the actual lesion morphology. As can be observed in the contrast between the original image and the segmentation mask, the model successfully identified the tumor region with hyperintensity in the T2w-FLAIR image and the lesion boundary after contrast enhancement in the T1-weighted image. In addition, the high activation regions shown by the heatmap highly coincide with the segmented regions, indicating that the model does pay attention to tumor-related imaging features during the decision-making process. This result not only validates the strong spatial localization ability of the model, but also demonstrates its ability to capture fine-grained features with diagnostic value, thereby significantly enhancing the interpretability of the model and its potential application in clinical scenarios. Compared with the visualization method based on classification prediction only, the segmentation results provide more explicit structural boundary information, so that the model's recognition of the lesion area is no longer limited to the fuzzy focus area, but has the practical ability to localize the lesion.

## 4 Discussion

We proposed a federated multi-task learning framework that enables collaborative training across multiple clinical centers without the need to share raw patient data. The method leverages preoperative multi-parametric MRI to simultaneously predict key molecular and histopathological features in newly diagnosed glioma patients, including IDH mutation, 1p/19q co-deletion, MGMT promoter methylation, and tumor grade, while also performing automated segmentation of T2w hyperintense regions. Departing from the classical FedAvg algorithm, our frame- work incorporates compressed encoding and auxiliary decoding mechanisms to accommodate inter-institutional label inconsistencies and data heterogeneity (29). For patients for whom re-resection is infeasible or only a biopsy can be obtained, this non-invasive approach could potentially serve as a supplementary adjunct, offering decision support in the presence of diagnostic uncertainty.

In the test set, our method achieved promising performance in predicting molecular and histological features, with AUCs of 0.88 for IDH, 0.84 for 1p/19q, 0.85 for MGMT, and 0.94 for tumor grade. Notably, the test data were not involved in model development or hyperparameter tuning, thus providing an initial assessment of generalizability. Leveraging advanced GPU hardware, we trained a large-scale deep learning model that directly ingests full 3D MRI volumes. Combined with a diverse, multi-center dataset, our approach demonstrated a degree of robustness to clinical imaging variability, suggesting potential for further investigation in real-world settings.

By integrating multi-task learning with federated training, our framework learns shared representations of molecular and histopathological features across institutions, while preserving data privacy. This joint modeling approach not only enhances the contextual consistency between predicted attributes (e.g., preventing biologically implausible co-occurrences such as IDH-wildtype with 1p/19q co-deletion) but also improves the model's generalization ability by exploiting inter-center heterogeneity (30). Unlike traditional classification systems based solely on fixed subtypes, our method independently predicts individual markers, allowing seamless compatibility with modern glioma classification guidelines, such as WHO 2021, and thus exhibits higher clinical adaptability (31).

Several existing studies have attempted to jointly predict glioma biomarkers using multi-task networks. Xu et al. proposed a model that simultaneously predicts multiple molecular indicators and the overall survival of GBM patients (32). However, this method was restricted to GBM and required prior tumor grading, limiting its utility in preoperative settings. Moreover, it relied on manual tumor segmentation, increasing clinical workload and deployment complexity. Similarly, Tupe-Waghmare et al. introduced a multi-task network that performs tumor segmentation and predicts both tumor grade (LGG vs. HGG) and IDH mutation status (33). However, their model lacked the ability to predict 1p/19q status, a limitation for complete WHO 2016 classification. Another related effort by Decuyper et al. developed a model to jointly predict IDH, 1p/19q, and tumor grade (34), but their approach required pre-segmented tumor masks obtained via a separate U-Net model, effectively relying on a dual-network pipeline. In contrast, our approach adopts a unified end-to-end architecture, avoiding the complexity and potential error accumulation from separate segmentation stages. A key aspect of our study is the evaluation on a completely independent external test set, which enhances the reliability of our findings.

Despite the promising results, this study has several important limitations that must be acknowledged. First, our model was developed and validated using publicly available, retrospective datasets. While these multi-center datasets provide diversity, they are often curated and may not fully represent the complexity and noise of prospective, real-world data encountered in a live clinical environment. The framework's performance in a true, operational federated learning network across hospitals with private, uncurated data remains to be validated.

Second, and critically, the model's performance was inconsistent across different molecular subtypes, particularly for rarer groups. For example, IDH-wildtype GBMs were predicted with high accuracy, whereas the model struggled with distinguishing IDH-mutant and 1p/19q-codeleted LGGs from other LGGs (35). This was particularly evident in Grade II tumors, where sensitivity was suboptimal. This performance gap is a significant limitation, likely stemming from the small number of samples for these rare subtypes in the training cohort (36). Although we applied imbalance-handling strategies during training, data diversity and subgroup size disparities still limited overall performance (37, 38).The federated framework allowed us to mitigate these limitations to some extent by enabling model training across multiple institutions, improving generalizability across diverse patient populations (39, 40).

Third, the practical deployment of federated learning presents substantial real-world challenges. Data heterogeneity (Non-IID) across centers, while a source of generalization, can also lead to inconsistent local model updates, potentially slowing convergence or causing performance fluctuations. Furthermore, the variability in data quality from different MRI scanners and protocols introduces potential biases that are difficult to fully mitigate without direct data access. Finally, the logistical and infrastructural hurdles are non-trivial. Our large 3D model necessitates significant communication overhead, requiring stable and high-bandwidth network infrastructure between participating institutions. Remote debugging and troubleshooting in a privacy-preserving setting are also far more complex than in a centralized environment.

In future work, we plan to incorporate advanced imaging modalities such as dynamic contrast-enhanced MRI (DCE-MRI) and MR spectroscopy (MRSI), which have shown potential in capturing tumor biology and treatment response. These modalities were excluded in this study due to their limited availability in routine clinical settings, in contrast to the conventional MRI sequences we used. While integrating DCE-MRI and MRSI may reduce data availability and model accessibility (41, 42), the growing adoption of these techniques in clinical practice may facilitate their inclusion in future studies and potentially enhance model accuracy. Future efforts must also focus on prospective validation within a real-world federated network to address the aforementioned challenges of infrastructure and data heterogeneity directly.

In conclusion, we have developed a federated learning-based multi-task approach that can jointly learn from multiple medical institutions to achieve the prediction of IDH mutation status, 1p/19q co-deletion status, MGMT methylation status and tumor grade without sharing the original data, and automatically complete the segmentation of tumor regions based on preoperative MRI images. The proposed method shows good generalization performance on three independent test datasets, and has real cross-center adaptability. The analysis strategy of simultaneously predicting multiple clinical indicators rather than relying on a single task is more in line with the actual needs of comprehensive evaluation of multiple diagnostic factors in clinical practice. In addition, this method does not rely on complex prior knowledge in the training phase and does not limit the applicable patient population, which helps to expand the clinical coverage.

## Data Availability

The original contributions presented in the study are included in the article/Supplementary Material, further inquiries can be directed to the corresponding authors.
